# Synthesis, crystal structure and Hirshfeld surface analysis of (2-amino-1-methyl­benzimidazole-κ*N*^3^)aqua­bis­(4-oxopent-2-en-2-olato-κ^2^*O*,*O*′)nickel(II) ethanol monosolvate

**DOI:** 10.1107/S2056989024008958

**Published:** 2024-10-22

**Authors:** Kyzlarkhan Siddikova, Murodov Sardor, Akmaljon Tojiboyev, Zukhra Kadirova, Jamshid Ashurov, Shahlo Daminova

**Affiliations:** a Karshi Engineering Economics Institute, Mustakillik Avenue, 225, Karshi 180100, Uzbekistan; b National University of Uzbekistan named after Mirzo Ulugbek University Street 4 Tashkent 100174 Uzbekistan; c Uzbekistan-Japan Innovation Centre of Youth, University Street 2B, Tashkent 100095, Uzbekistan; d University of Geological Sciences, Olimlar Street, 64, Tashkent 100170, Uzbekistan; e Institute of Bioorganic Chemistry, Academy of Sciences of Uzbekistan, Mirzo, Ulugbek Street 83, Tashkent 100125, Uzbekistan; Universidad de la Repüblica, Uruguay

**Keywords:** crystal structure, mixed-ligand complex, hydrogen bonding, benzimidazole, acetyl­acetone, Hirshfeld surface

## Abstract

The mol­ecule of the title compound, [Ni(C_5_H_7_O_2_)_2_(C_8_H_9_N_3_)(H_2_O)]·C_2_H_5_OH, has triclinic (*P*) symmetry. This compound is of inter­est for its anti­microbial properties. The asymmetric unit comprises two independent complex mol­ecules, which are linked by N—H⋯O and O—H⋯O hydrogen bonds along [111]. Hirshfeld surface analysis indicates that 71.7% of inter­mol­ecular inter­actions come from H⋯H contacts, 17.7% from C⋯H/H⋯C contacts and 7.6% from O⋯H/H⋯O contacts, with the remaining contribution coming from N⋯H/H⋯N, C⋯N/N⋯C, C⋯C and O⋯O contacts.

## Abstract 2

The title compound was synthesized from acetyl­acetone and benzimidazole derivative. There are two independent complex mol­ecules in the asymmetric unit, which are linked by N—H⋯O and O—H⋯O hydrogen bonds along [111].

## 1. Chemical context

β-Di­carbonyl compounds are widely known for their keto–enol equilibria and are the leading tautomeric systems studied (Tighadouini *et al.*, 2022 ▸; Thomas, 2001 ▸). Acet­ylacetonate (acac), as the most representative example, forms strong coordination compounds in which both oxygen atoms coordinate with the metal and form four- and six-membered chelate complexes (Smith *et al.*, 2016 ▸; Zheleznova *et al.*, 2021 ▸). It is used in analytics as a bidentate ligand for the determination of *d*-metals (Co, Mn, Fe, Ni, Cu), and in radiochemistry for the isolation of radioisotopes (Caminati & Grabow, 2006 ▸). Complexes of rare earth atoms with β-diketonates have been widely studied due to the ease of use of diketonates as organic ligands (Binnemans, 2005 ▸; Duan *et al.*, 2022 ▸). These ligands can increase the efficiency and intensity of luminescence, one such complex being Eu(acac)_3_ (Kuzmina & Eliseeva, 2006 ▸). In addition, Tb(acac)_3_ is used as the active light-emitting layer in the first LEDs based on lanthanide complexes (Kido *et al.*, 1990 ▸). Benzimidazole derivatives are an important class of heteroaromatic compounds due to their biological and pharmaceutical activities (Keri *et al.*, 2015 ▸; Pathare *et al.*, 2021 ▸). The benzimidazole unit has seven positions for substitution of various moieties. Most bioactive compounds based on benzimidazole derivatives bearing functional groups at positions 1, 2 and/or 5 (or 6) have been described in the literature (Bansal & Silakari, 2012 ▸). A large number of benzimidazole derivatives has been found to have anti­bacterial (Elnima *et al.*, 1981 ▸; Ablo *et al.*, 2023 ▸), anti­viral (Townsend *et al.*, 1995 ▸; Marinescu, 2023 ▸), anti­fungal (Desai & Desai, 2006 ▸; Morcoss *et al.*, 2023 ▸), anti­asthmatic (Ramanatham *et al.*, 2008 ▸), anti-HIV (Li *et al.*, 2009 ▸; Kabi *et al.*, 2022 ▸), anti­convulsant (Bhrigu *et al.*, 2012 ▸; Shabana *et al.*, 2023 ▸), anti­hypertensive (Jain *et al.*, 2013 ▸; Tajane *et al.*, 2022 ▸) and anti­depressant (Mathew *et al.*, 2016 ▸) activities. In this regard, we synthesized the title compound (**I**) for feedstocks with anti­microbial properties. This study presents its structural characterization and investigation of its three-dimensional structure, including investigation of hydrogen-bond strength and Hirshfeld surface analysis.
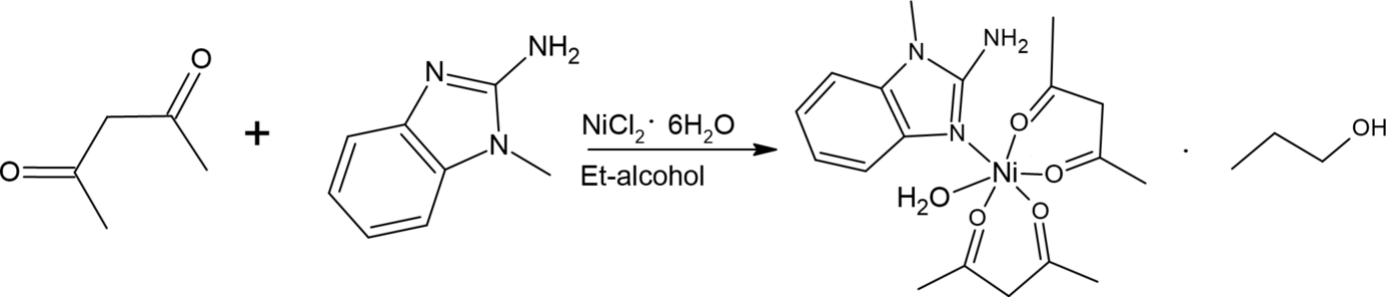


## 2. Structural commentary

**Figure 1:**
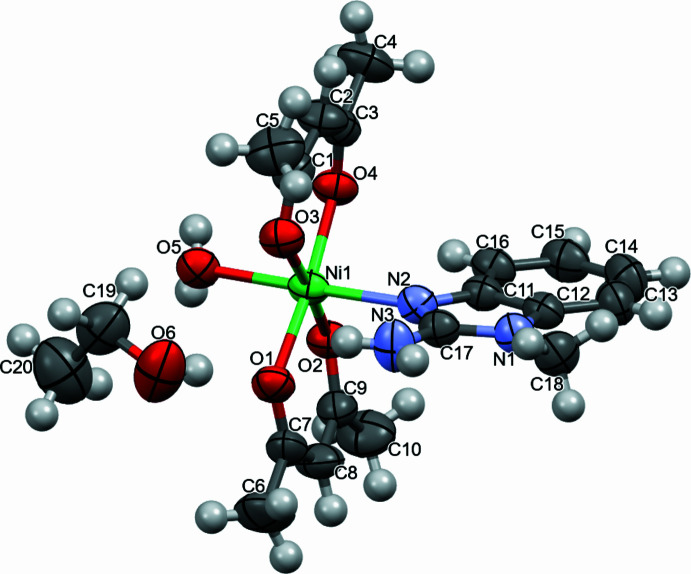
Asymmetric unit of the title compound with the atom-numbering scheme. Displacement ellipsoids for non-hydrogen atoms are drawn at the 50% probability level.

**Figure 2:**
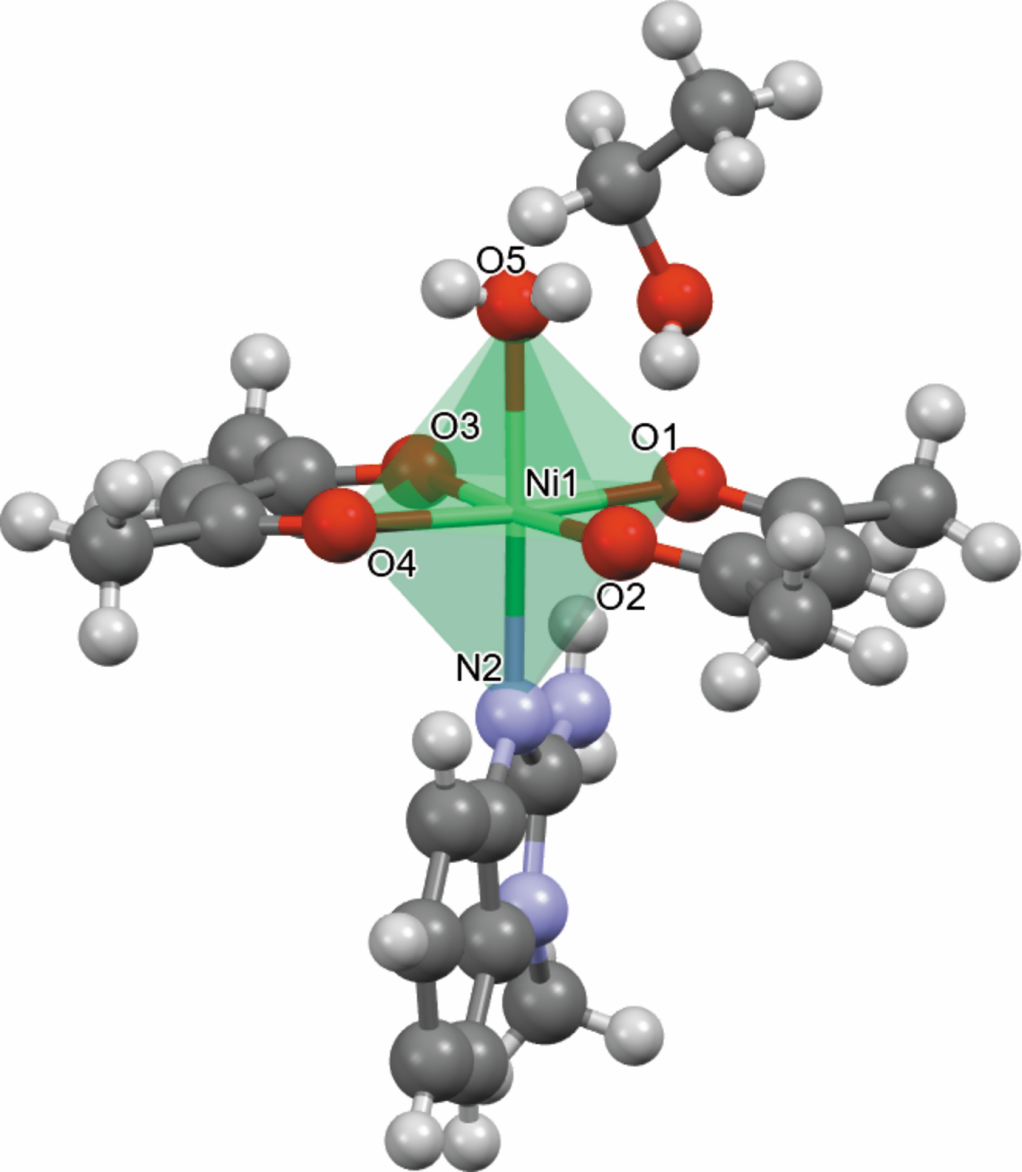
Representation of the octahedral coordination sphere around the metal centre in the title compound.

The title compound **I** (Fig. 1 ▸) crystallizes in the triclinic system in the space group *P*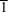. This heteroligand complex is composed of two ligands of the β-dikeonide type and a benzimidazole derivative. The asymmetric unit consists of two acet­ylacetonate (acac), one 2-amino-1-methlybenzimidazole (MAB) ligand and a water mol­ecule that are coordinated with nickel, and one ethanol mol­ecule. In complex **I**, the Ni atom has a coordination number of six. The acac ligands act as bidentate ligands, coordinating to the central nickel atom via the oxygen atoms of their carbonyl groups. One coordination bond is formed due to the benzimidazole ring, where coordination occurs through the *sp*^2^ nitro­gen heteroatom, which is located in the five-membered ring of the ligand. The other coordination is formed due to the O atom of the water mol­ecule. The Ni atom displays an octahedral geometry (Fig. 2 ▸); the axial positions are occupied by atom N2 of the five-membered ring of the benzimidazole ligand and by the water oxygen atom O5, with an N2—Ni1—O5 angle of 178.99 (7)(9)°. The equatorial plane is formed by β-diketonide oxygen atoms. The O1–O4/Ni1 plane has an r.m.s. deviation of 0.030 Å, with an out-of-plane distance of 0.0559 (4) Å for Ni. The large variation in the bond angles at nickel is due to the bidentate acac ligands (Table 1 ▸). The closeness of the values for the O1—Ni1—O2 and O3—Ni1—O4 and for the O1—Ni1—O4 and O2—Ni1—O3 angles is explained by the presence of a hydrogen bond on one side of the complex.

## 3. Supra­molecular features

**Figure 3:**
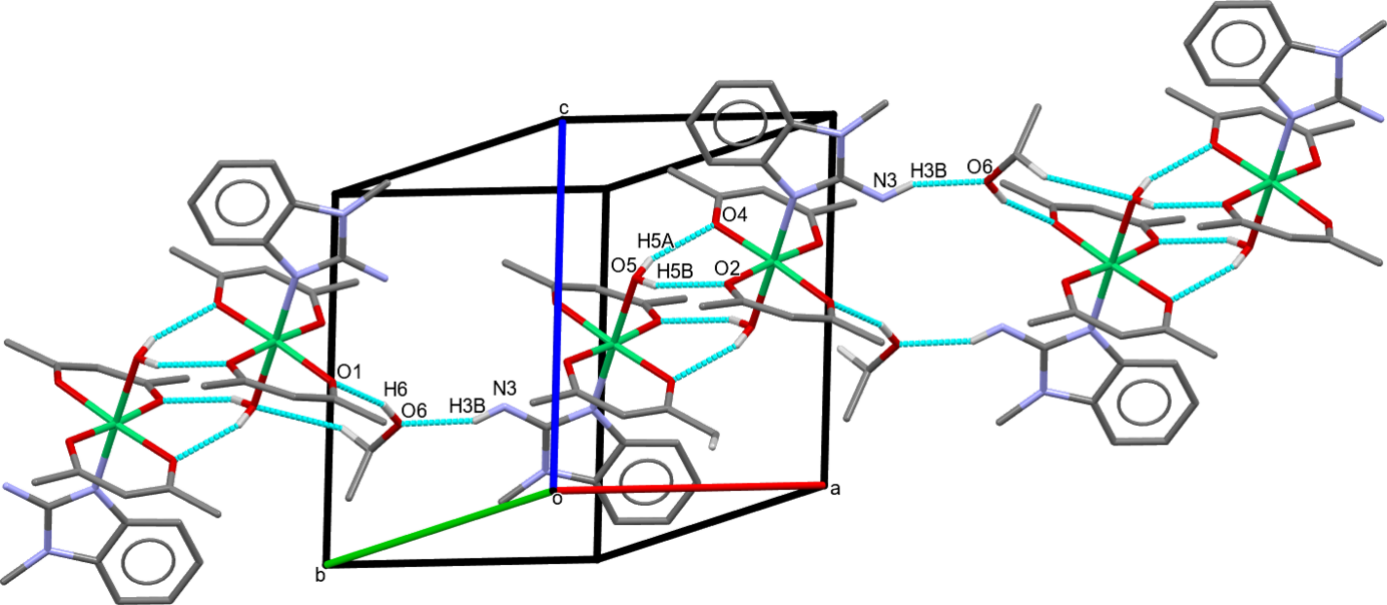
Packing diagram of **I** showing the N—H⋯O and O—H⋯O hydrogen bonding resulting in chains along [111]. Only H atoms involved in the inter­actions are shown.

N—H⋯O and O—H⋯O hydrogen bonds (Table 2 ▸) are observed in the crystal. The O6—H6⋯O1, N3—H3*B*⋯O6, O5—H5*A*⋯O4 and O5—H5*B*⋯O2 hydrogen bonds link the complex mol­ecules into chains along the [111] direction (Fig. 3 ▸). The co-crystallized ethanol mol­ecule is linked with an acac oxygen atom by the O6—H6⋯O1 hydrogen bond, and with a benzimidazole nitro­gen atom of a neighbouring mol­ecule by the N3—H3*B*⋯O6 hydrogen bond.

## 4. Hirshfeld surface

**Figure 4:**
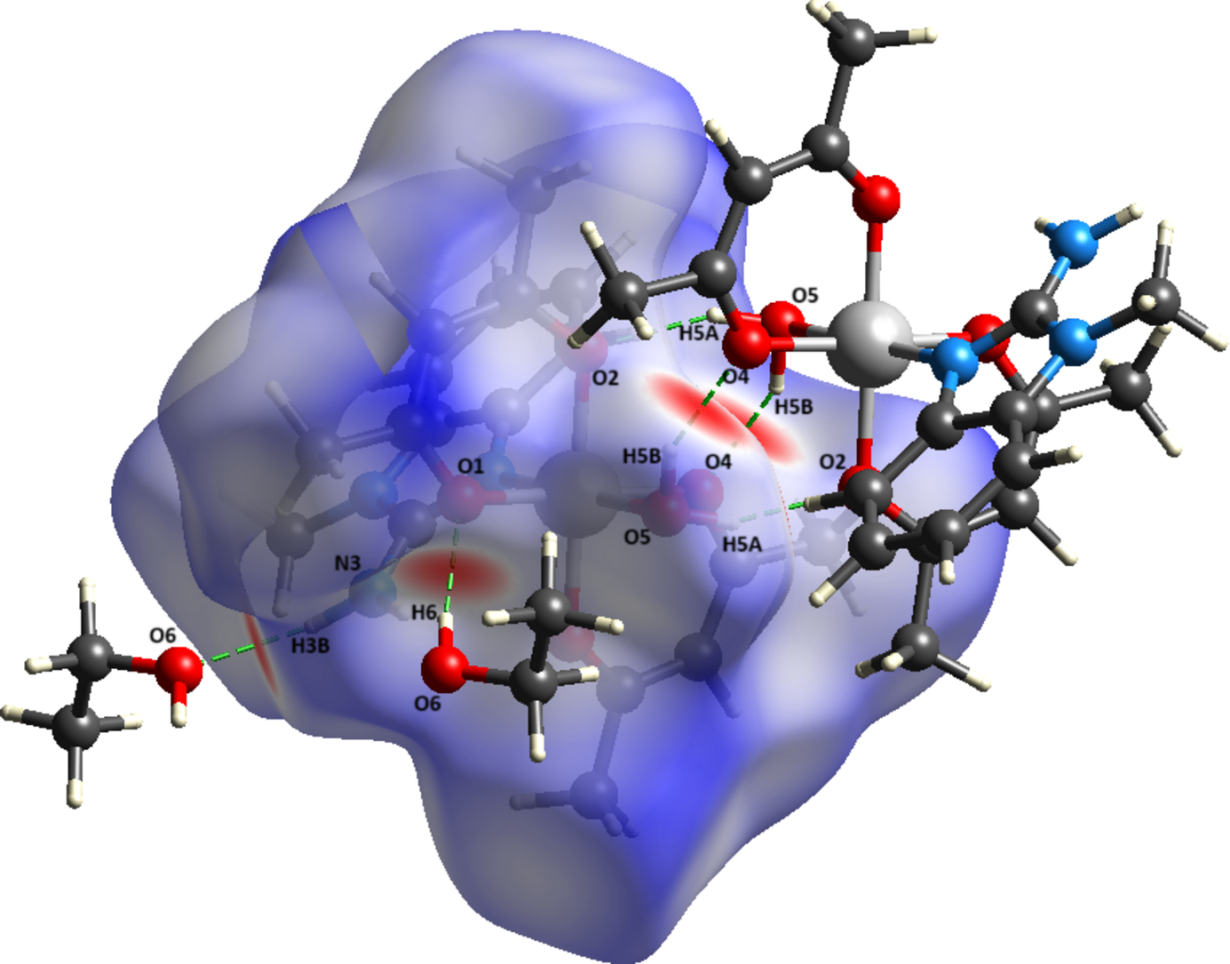
Hirshfeld surface of **I** mapped over <*d*_norm_ showing close inter­mol­ecular contacts.

**Figure 5:**
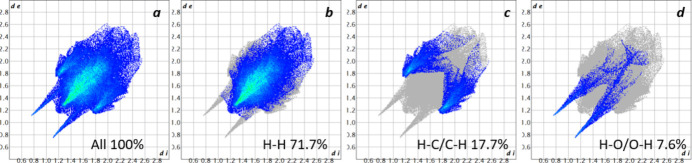
(*a*) The full two-dimensional fingerprint plots for the title compound, showing all inter­actions and (*b–d*) those delineated into specified inter­actions.

A Hirshfeld surface analysis (HS) was performed using *Crystal Explorer 21.5* (Spackman *et al.*, 2021 ▸). On the HS plotted over *d*_norm_ (Fig. 4 ▸), white areas indicates contacts with distances equal to the sum of the van der Waals radii, while red and blue areas indicate distances shorter (in close contact) or longer (distant contact), respectively, than the van der Waals radii (Venkatesan *et al.*, 2016 ▸). The overall 2D fingerprint plot is shown in Fig. 5 ▸*a*. The largest contribution to the Hirshfeld surface is made the H⋯H contacts(Fig. 5 ▸*b*), which account for 71.7%. H⋯C/C⋯H (Fig. 5 ▸*c*) and O⋯H/H⋯O (Fig. 5 ▸*d*) contacts contribute 17.7% and 7.6%, respectively. The remaining contributions are from N⋯H/H⋯N, C⋯N/N⋯C, C⋯C and O⋯O contacts (2.2%, 0.6%, 0.1% and 0.1%, respectively).

## 5. Database survey

A search of the Cambridge Structural Database (CSD2023.2.0, version 5.45, November 2023; Groom *et al.*, 2016 ▸) revealed three similar structures with fragment **I.** In particular, structures including nickel complexes with the acac ligand have been described [refcodes: ACNIPC (Anzenhofer & Hewitt, 1971 ▸), ACNIPC01 (Cramer *et al.*, 1977 ▸) and HOWSIX (Hämmerling *et al.*, 2018 ▸). In one study with a fragment including MAB, the anti­microbial properties of the ligand itself with different metals were studied (LUNCIH; de Jongh *et al.*, 2009 ▸).

## 6. Synthesis and crystallization

Preparation of solutions: (*a*) ethanol solution of 0.1 mmol (0.0238 g) of NiCl_2_·6H_2_O, (*b*) ethanol solution of 0.2 mmol (0.0294 g) of MAB and (*c*) acac (0.2 mmol; *V* = 0.0205 ml, ρ = 0.975 g ml^−1^). Solution *a* was added to solution *b* and stirred for 30 minutes at room temperature on a magnetic stirrer. After this, solution *c* was added dropwise and stirred for 12 h, during which time it turned yellow. After several days, a yellow precipitate formed, which was filtered and washed several times with ethanol. Since the primary sediment, as well as the resulting crystals, can be dissolved in DMF and DMSO, recrystallization was carried out in DMF. After the recrystallization process, light-yellow single crystals were obtained.

## 7. Refinement details

Crystal data, data collection and structure refinement details are summarized in (Table 3 ▸). C-bound H atoms were positioned geometrically and treated as riding on their parent atoms, with C—H = 0.93 Å (aromatic), 0.96 Å (meth­yl) or 0.97 Å (methyl­ene) and were refined with *U*_iso_(H) = 1.5*U*_eq_(C) for methyl H atoms and 1.2U_*eq*_(C) otherwise. The hy­droxy H atom was positioned with an O—H = 0.84 Å and water O atoms with O—H = 0.82 Å and refined with *U*_iso_(H) = 1.5*U*_eq_(O).

## Supplementary Material

Crystal structure: contains datablock(s) I. DOI: 10.1107/S2056989024008958/ny2006sup1.cif

Structure factors: contains datablock(s) I. DOI: 10.1107/S2056989024008958/ny2006Isup2.hkl

CCDC reference: 2383609

Additional supporting information:  crystallographic information; 3D view; checkCIF report

## Figures and Tables

**Table 1:** Selected geometric parameters (Å, °)

Ni1—O1	2.0273 (18)	Ni1—O4	2.0353 (17)
Ni1—O2	2.0286 (17)	Ni1—O5	2.1303 (18)
Ni1—O3	2.0110 (18)	Ni1—N2	2.082 (2)
			
O1—Ni1—O2	89.60 (8)	O3—Ni1—O4	90.29 (7)
O1—Ni1—O4	175.17 (7)	N2—Ni1—O5	178.99 (7)
O3—Ni1—O2	176.18 (8)		

**Table 2:** Hydrogen-bond geometry (Å, °)

*D*—H⋯*A*	*D*—H	H⋯*A*	*D*⋯*A*	*D*—H⋯*A*
N3—H3*B*⋯O6^i^	0.86	2.09	2.897 (3)	156
O5—H5*A*⋯O4^ii^	0.85	1.99	2.773 (3)	152
O5—H5*B*⋯O2^ii^	0.96	1.86	2.777 (3)	160
O6—H6⋯O1	0.82	2.01	2.811 (4)	166

Symmetry codes: (i) 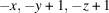; (ii) 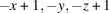.

**Table 3:** Experimental details

Crystal data	
Chemical formula	[Ni(C_5_H_7_O_2_)_2_(C_8_H_9_N_3_)(H_2_O)]·C_2_H_6_O
*M*_r_	468.19
Crystal system, space group	Triclinic, *P* 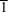
Temperature (K)	293
*a*, *b*, *c* (Å)	10.6348 (3), 11.1390 (4), 11.7989 (3)
α, β, γ (°)	72.392 (3), 64.047 (3), 75.829 (3)
*V* (Å^3^)	1187.52 (7)
*Z*	2
Radiation type	Cu *K*α
μ (mm^−1^)	1.50
Crystal size (mm)	0.2 × 0.2 × 0.1

Data collection	
Diffractometer	Rigaku XtaLAB Synergy (Single source at home/near) diffractometer with a HyPix3000 detector
Absorption correction	Multi-scan (*CrysAlis PRO*; Rigaku OD, 2020 ▸)
*T*_min_, *T*_max_	0.731, 1.000
No. of measured, independent and observed [*I* ≥ 2u(*I*)] reflections	11590, 4502, 3361
*R*_int_	0.040
(sin θ/λ)_max_ (Å^−1^)	0.609

Refinement	
*R*[*F*^2^ > 2σ(*F*^2^)], *wR*(*F*^2^), *S*	0.048, 0.139, 1.08
No. of reflections	4502
No. of parameters	280
H-atom treatment	H-atom parameters constrained
Δρ_max_, Δρ_min_ (e Å^−3^)	0.24, −0.33

Computer programs: *CrysAlis PRO* (Rigaku OD, 2020 ▸), *OLEX2.solve* (Bourhis *et al.*, 2015 ▸), *SHELXL2018/3* (Sheldrick, 2015 ▸) and *OLEX2* (Dolomanov *et al.*, 2009 ▸).

## supplementary crystallographic information

### (2-Amino-1-methylbenzimidazole-κN3)aquabis(4-oxopent-2-en-2-olato-κ2O,O')nickel(II) ethanol monosolvate Crystal data

**Table:**

[Ni(C_5_H_7_O_2_)_2_(C_8_H_9_N_3_)(H_2_O)]·C_2_H_6_O	*Z* = 2
*M**_r_* = 468.19	*F*(000) = 496
Triclinic, *P*1	*D*_x_ = 1.309 Mg m^−^^3^
*a* = 10.6348 (3) Å	Cu *K*α radiation, λ = 1.54184 Å
*b* = 11.1390 (4) Å	Cell parameters from 3401 reflections
*c* = 11.7989 (3) Å	θ = 4.2–69.5°
α = 72.392 (3)°	µ = 1.50 mm^−^^1^
β = 64.047 (3)°	*T* = 293 K
γ = 75.829 (3)°	Rhombohedral, clear yellowish yellow
*V* = 1187.52 (7) Å^3^	0.2 × 0.2 × 0.1 mm

### (2-Amino-1-methylbenzimidazole-κN3)aquabis(4-oxopent-2-en-2-olato-κ2O,O')nickel(II) ethanol monosolvate Data collection

**Table:**

Rigaku XtaLAB Synergy (Single source at home/near) diffractometer with a HyPix3000 detector	4502 independent reflections
Radiation source: micro-focus sealed X-ray tube, PhotonJet (Cu) X-ray Source	3361 reflections with *I*≥ 2u(*I*)
Mirror monochromator	*R*_int_ = 0.040
Detector resolution: 10.0000 pixels mm^-1^	θ_max_ = 70.0°, θ_min_ = 4.2°
ω scans	*h* = −12→12
Absorption correction: multi-scan (CrysAlisPro; Rigaku OD, 2020)	*k* = −13→11
*T*_min_ = 0.731, *T*_max_ = 1.000	*l* = −14→14
11590 measured reflections	

### (2-Amino-1-methylbenzimidazole-κN3)aquabis(4-oxopent-2-en-2-olato-κ2O,O')nickel(II) ethanol monosolvate Refinement

**Table:**

Refinement on *F*^2^	45 constraints
Least-squares matrix: full	Primary atom site location: anomalous-dispersion techniques
*R*[*F*^2^ > 2σ(*F*^2^)] = 0.048	Hydrogen site location: mixed
*wR*(*F*^2^) = 0.139	H-atom parameters constrained
*S* = 1.08	*w* = 1/[σ^2^(*F*_o_^2^) + (0.0661*P*)^2^] where *P* = (*F*_o_^2^ + 2*F*_c_^2^)/3
4502 reflections	(Δ/σ)_max_ = 0.001
280 parameters	Δρ_max_ = 0.24 e Å^−^^3^
0 restraints	Δρ_min_ = −0.33 e Å^−^^3^

### (2-Amino-1-methylbenzimidazole-κN3)aquabis(4-oxopent-2-en-2-olato-κ2O,O')nickel(II) ethanol monosolvate Special details

**Table:**

Geometry. All esds (except the esd in the dihedral angle between two l.s. planes) are estimated using the full covariance matrix. The cell esds are taken into account individually in the estimation of esds in distances, angles and torsion angles; correlations between esds in cell parameters are only used when they are defined by crystal symmetry. An approximate (isotropic) treatment of cell esds is used for estimating esds involving l.s. planes.

### (2-Amino-1-methylbenzimidazole-κN3)aquabis(4-oxopent-2-en-2-olato-κ2O,O')nickel(II) ethanol monosolvate Fractional atomic coordinates and isotropic or equivalent isotropic displacement parameters (Å^2^)

**Table:**

	*x*	*y*	*z*	*U*_iso_*/*U*_eq_	
Ni1	0.37833 (4)	0.20417 (4)	0.43078 (4)	0.04770 (17)	
O1	0.24815 (19)	0.30620 (17)	0.56482 (18)	0.0569 (5)	
O2	0.53607 (18)	0.19252 (16)	0.48789 (18)	0.0537 (4)	
O3	0.21418 (18)	0.21047 (17)	0.38640 (19)	0.0572 (5)	
O4	0.50040 (18)	0.08899 (16)	0.30636 (18)	0.0536 (4)	
O5	0.31402 (17)	0.03642 (16)	0.57499 (17)	0.0553 (5)	
H5A	0.360544	0.020598	0.622452	0.083*	
H5B	0.354854	−0.037802	0.539752	0.083*	
N1	0.4363 (2)	0.5612 (2)	0.1599 (2)	0.0541 (5)	
N2	0.4427 (2)	0.36623 (19)	0.2874 (2)	0.0497 (5)	
N3	0.2214 (2)	0.4934 (2)	0.3222 (2)	0.0673 (7)	
H3A	0.177127	0.435389	0.384603	0.081*	
H3B	0.175491	0.564935	0.299385	0.081*	
C1	0.2180 (3)	0.1699 (2)	0.2975 (3)	0.0552 (7)	
C2	0.3363 (3)	0.1014 (3)	0.2183 (3)	0.0668 (8)	
H2	0.326316	0.079068	0.153114	0.080*	
C3	0.4668 (3)	0.0634 (2)	0.2273 (3)	0.0549 (7)	
C4	0.5806 (4)	−0.0174 (3)	0.1394 (3)	0.0810 (10)	
H4A	0.650140	0.034189	0.072629	0.121*	
H4B	0.539541	−0.052993	0.100909	0.121*	
H4C	0.624512	−0.084707	0.188752	0.121*	
C5	0.0829 (4)	0.1984 (4)	0.2761 (4)	0.0879 (11)	
H5C	0.014455	0.147918	0.346155	0.132*	
H5D	0.100950	0.178503	0.196177	0.132*	
H5E	0.047252	0.286823	0.272283	0.132*	
C6	0.1747 (4)	0.4474 (4)	0.7014 (4)	0.1009 (13)	
H6A	0.118362	0.499179	0.654862	0.151*	
H6B	0.217212	0.500831	0.721237	0.151*	
H6C	0.116146	0.396497	0.780293	0.151*	
C7	0.2884 (3)	0.3620 (3)	0.6197 (3)	0.0603 (7)	
C8	0.4254 (3)	0.3492 (3)	0.6101 (3)	0.0706 (8)	
H8	0.442871	0.401686	0.648294	0.085*	
C9	0.5403 (3)	0.2655 (3)	0.5491 (3)	0.0566 (7)	
C10	0.6783 (4)	0.2561 (4)	0.5586 (4)	0.0921 (12)	
H10A	0.693330	0.177588	0.616418	0.138*	
H10B	0.676903	0.325993	0.590987	0.138*	
H10C	0.753214	0.258691	0.474542	0.138*	
C11	0.5795 (3)	0.3873 (2)	0.1982 (2)	0.0500 (6)	
C12	0.5768 (3)	0.5081 (3)	0.1180 (3)	0.0528 (6)	
C13	0.6961 (3)	0.5544 (3)	0.0182 (3)	0.0667 (8)	
H13	0.691386	0.634632	−0.035448	0.080*	
C14	0.8224 (3)	0.4763 (3)	0.0018 (3)	0.0745 (9)	
H14	0.905466	0.504785	−0.063062	0.089*	
C15	0.8272 (3)	0.3549 (3)	0.0815 (3)	0.0688 (8)	
H15	0.913756	0.303874	0.067631	0.083*	
C16	0.7076 (3)	0.3086 (3)	0.1800 (3)	0.0575 (7)	
H16	0.712227	0.227718	0.232527	0.069*	
C17	0.3614 (3)	0.4725 (2)	0.2605 (3)	0.0519 (6)	
C18	0.3795 (3)	0.6842 (3)	0.1015 (3)	0.0680 (8)	
H18A	0.382641	0.748120	0.139153	0.102*	
H18B	0.283564	0.682305	0.116713	0.102*	
H18C	0.434695	0.703474	0.010108	0.102*	
O6	−0.0365 (2)	0.2675 (2)	0.6737 (3)	0.1025 (9)	
H6	0.045101	0.281874	0.629680	0.154*	
C19	−0.0391 (4)	0.1356 (4)	0.7187 (4)	0.0948 (12)	
H19A	0.029258	0.093414	0.650879	0.100 (12)*	
H19B	−0.131701	0.116749	0.738011	0.139 (17)*	
C20	−0.0070 (5)	0.0844 (5)	0.8363 (5)	0.1266 (17)	
H20A	0.087857	0.095682	0.815678	0.190*	
H20B	−0.016667	−0.004499	0.866881	0.190*	
H20C	−0.071434	0.128771	0.902373	0.190*	

### (2-Amino-1-methylbenzimidazole-κN3)aquabis(4-oxopent-2-en-2-olato-κ2O,O')nickel(II) ethanol monosolvate Atomic displacement parameters (Å^2^)

**Table:**

	*U*^11^	*U*^22^	*U*^33^	*U*^12^	*U*^13^	*U*^23^
Ni1	0.0429 (3)	0.0473 (3)	0.0525 (3)	0.00597 (18)	−0.0188 (2)	−0.0200 (2)
O1	0.0500 (10)	0.0551 (10)	0.0619 (12)	0.0005 (8)	−0.0140 (9)	−0.0263 (9)
O2	0.0512 (10)	0.0534 (10)	0.0602 (11)	0.0029 (8)	−0.0258 (9)	−0.0195 (9)
O3	0.0458 (10)	0.0592 (11)	0.0667 (12)	0.0037 (8)	−0.0218 (9)	−0.0234 (9)
O4	0.0522 (10)	0.0525 (10)	0.0591 (11)	0.0094 (8)	−0.0254 (9)	−0.0241 (9)
O5	0.0501 (10)	0.0512 (10)	0.0599 (11)	0.0060 (8)	−0.0218 (9)	−0.0160 (8)
N1	0.0502 (12)	0.0509 (12)	0.0544 (13)	0.0003 (9)	−0.0189 (10)	−0.0104 (10)
N2	0.0399 (11)	0.0505 (12)	0.0542 (13)	0.0038 (9)	−0.0170 (10)	−0.0159 (10)
N3	0.0431 (12)	0.0567 (14)	0.0758 (17)	0.0081 (10)	−0.0136 (12)	−0.0053 (12)
C1	0.0545 (16)	0.0496 (14)	0.0662 (18)	−0.0040 (12)	−0.0300 (14)	−0.0119 (13)
C2	0.0696 (19)	0.079 (2)	0.0680 (19)	0.0018 (15)	−0.0373 (16)	−0.0318 (16)
C3	0.0602 (17)	0.0495 (14)	0.0503 (15)	0.0015 (12)	−0.0202 (13)	−0.0143 (12)
C4	0.084 (2)	0.091 (2)	0.070 (2)	0.0163 (18)	−0.0302 (18)	−0.0430 (19)
C5	0.071 (2)	0.095 (3)	0.119 (3)	0.0038 (18)	−0.055 (2)	−0.037 (2)
C6	0.090 (3)	0.101 (3)	0.109 (3)	0.006 (2)	−0.015 (2)	−0.071 (2)
C7	0.0689 (18)	0.0498 (15)	0.0539 (16)	−0.0055 (13)	−0.0120 (14)	−0.0206 (13)
C8	0.075 (2)	0.075 (2)	0.076 (2)	−0.0103 (16)	−0.0279 (17)	−0.0388 (17)
C9	0.0608 (17)	0.0592 (16)	0.0550 (16)	−0.0134 (13)	−0.0242 (13)	−0.0132 (13)
C10	0.077 (2)	0.117 (3)	0.110 (3)	−0.011 (2)	−0.049 (2)	−0.046 (3)
C11	0.0466 (14)	0.0550 (14)	0.0501 (15)	−0.0007 (11)	−0.0191 (12)	−0.0188 (12)
C12	0.0491 (14)	0.0606 (16)	0.0496 (15)	−0.0029 (12)	−0.0188 (12)	−0.0179 (12)
C13	0.0623 (18)	0.0702 (19)	0.0566 (17)	−0.0103 (15)	−0.0145 (14)	−0.0114 (15)
C14	0.0528 (17)	0.092 (2)	0.065 (2)	−0.0113 (16)	−0.0086 (14)	−0.0195 (18)
C15	0.0453 (16)	0.087 (2)	0.073 (2)	0.0046 (14)	−0.0183 (14)	−0.0341 (17)
C16	0.0462 (15)	0.0662 (17)	0.0586 (17)	0.0037 (12)	−0.0195 (13)	−0.0224 (14)
C17	0.0457 (14)	0.0512 (14)	0.0568 (16)	0.0009 (11)	−0.0191 (12)	−0.0170 (12)
C18	0.0701 (19)	0.0532 (16)	0.072 (2)	0.0045 (14)	−0.0291 (16)	−0.0115 (14)
O6	0.0585 (14)	0.0672 (15)	0.143 (2)	0.0080 (11)	−0.0163 (15)	−0.0190 (15)
C19	0.069 (2)	0.083 (3)	0.123 (3)	0.0004 (19)	−0.027 (2)	−0.035 (2)
C20	0.120 (4)	0.131 (4)	0.105 (4)	0.010 (3)	−0.043 (3)	−0.016 (3)

### (2-Amino-1-methylbenzimidazole-κN3)aquabis(4-oxopent-2-en-2-olato-κ2O,O')nickel(II) ethanol monosolvate Geometric parameters (Å, º)

**Table:**

Ni1—O1	2.0273 (18)	C6—H6B	0.9600
Ni1—O2	2.0286 (17)	C6—H6C	0.9600
Ni1—O3	2.0110 (18)	C6—C7	1.505 (4)
Ni1—O4	2.0353 (17)	C7—C8	1.385 (4)
Ni1—O5	2.1303 (18)	C8—H8	0.9300
Ni1—N2	2.082 (2)	C8—C9	1.400 (4)
O1—C7	1.268 (3)	C9—C10	1.497 (4)
O2—C9	1.259 (3)	C10—H10A	0.9600
O3—C1	1.244 (3)	C10—H10B	0.9600
O4—C3	1.257 (3)	C10—H10C	0.9600
O5—H5A	0.8541	C11—C12	1.393 (4)
O5—H5B	0.9587	C11—C16	1.395 (3)
N1—C12	1.387 (3)	C12—C13	1.382 (4)
N1—C17	1.366 (3)	C13—H13	0.9300
N1—C18	1.451 (3)	C13—C14	1.379 (4)
N2—C11	1.399 (3)	C14—H14	0.9300
N2—C17	1.327 (3)	C14—C15	1.398 (4)
N3—H3A	0.8600	C15—H15	0.9300
N3—H3B	0.8600	C15—C16	1.378 (4)
N3—C17	1.337 (3)	C16—H16	0.9300
C1—C2	1.404 (4)	C18—H18A	0.9600
C1—C5	1.508 (4)	C18—H18B	0.9600
C2—H2	0.9300	C18—H18C	0.9600
C2—C3	1.389 (4)	O6—H6	0.8200
C3—C4	1.511 (4)	O6—C19	1.405 (4)
C4—H4A	0.9600	C19—H19A	0.9700
C4—H4B	0.9600	C19—H19B	0.9700
C4—H4C	0.9600	C19—C20	1.486 (6)
C5—H5C	0.9600	C20—H20A	0.9600
C5—H5D	0.9600	C20—H20B	0.9600
C5—H5E	0.9600	C20—H20C	0.9600
C6—H6A	0.9600		
			
O1—Ni1—O2	89.60 (8)	C7—C6—H6C	109.5
O1—Ni1—O4	175.17 (7)	O1—C7—C6	115.4 (3)
O1—Ni1—O5	88.09 (7)	O1—C7—C8	125.1 (3)
O1—Ni1—N2	92.89 (8)	C8—C7—C6	119.5 (3)
O2—Ni1—O4	91.55 (7)	C7—C8—H8	116.6
O2—Ni1—O5	88.73 (7)	C7—C8—C9	126.9 (3)
O2—Ni1—N2	91.52 (8)	C9—C8—H8	116.6
O3—Ni1—O1	88.29 (8)	O2—C9—C8	124.5 (3)
O3—Ni1—O2	176.18 (8)	O2—C9—C10	115.9 (3)
O3—Ni1—O4	90.29 (7)	C8—C9—C10	119.5 (3)
O3—Ni1—O5	88.03 (7)	C9—C10—H10A	109.5
O3—Ni1—N2	91.76 (8)	C9—C10—H10B	109.5
O4—Ni1—O5	87.25 (7)	C9—C10—H10C	109.5
O4—Ni1—N2	91.77 (8)	H10A—C10—H10B	109.5
N2—Ni1—O5	178.99 (7)	H10A—C10—H10C	109.5
C7—O1—Ni1	124.91 (18)	H10B—C10—H10C	109.5
C9—O2—Ni1	125.12 (17)	C12—C11—N2	109.9 (2)
C1—O3—Ni1	126.34 (17)	C12—C11—C16	119.6 (3)
C3—O4—Ni1	125.35 (16)	C16—C11—N2	130.5 (2)
Ni1—O5—H5A	107.3	N1—C12—C11	105.6 (2)
Ni1—O5—H5B	111.2	C13—C12—N1	131.4 (3)
H5A—O5—H5B	96.5	C13—C12—C11	123.0 (3)
C12—N1—C18	126.1 (2)	C12—C13—H13	121.5
C17—N1—C12	107.0 (2)	C14—C13—C12	116.9 (3)
C17—N1—C18	126.8 (2)	C14—C13—H13	121.5
C11—N2—Ni1	128.07 (15)	C13—C14—H14	119.6
C17—N2—Ni1	127.06 (18)	C13—C14—C15	120.8 (3)
C17—N2—C11	104.9 (2)	C15—C14—H14	119.6
H3A—N3—H3B	120.0	C14—C15—H15	119.0
C17—N3—H3A	120.0	C16—C15—C14	122.0 (3)
C17—N3—H3B	120.0	C16—C15—H15	119.0
O3—C1—C2	125.1 (2)	C11—C16—H16	121.2
O3—C1—C5	115.7 (3)	C15—C16—C11	117.6 (3)
C2—C1—C5	119.1 (3)	C15—C16—H16	121.2
C1—C2—H2	116.6	N2—C17—N1	112.6 (2)
C3—C2—C1	126.7 (3)	N2—C17—N3	125.1 (3)
C3—C2—H2	116.6	N3—C17—N1	122.2 (2)
O4—C3—C2	125.2 (2)	N1—C18—H18A	109.5
O4—C3—C4	114.8 (2)	N1—C18—H18B	109.5
C2—C3—C4	120.0 (3)	N1—C18—H18C	109.5
C3—C4—H4A	109.5	H18A—C18—H18B	109.5
C3—C4—H4B	109.5	H18A—C18—H18C	109.5
C3—C4—H4C	109.5	H18B—C18—H18C	109.5
H4A—C4—H4B	109.5	C19—O6—H6	109.5
H4A—C4—H4C	109.5	O6—C19—H19A	109.1
H4B—C4—H4C	109.5	O6—C19—H19B	109.1
C1—C5—H5C	109.5	O6—C19—C20	112.6 (4)
C1—C5—H5D	109.5	H19A—C19—H19B	107.8
C1—C5—H5E	109.5	C20—C19—H19A	109.1
H5C—C5—H5D	109.5	C20—C19—H19B	109.1
H5C—C5—H5E	109.5	C19—C20—H20A	109.5
H5D—C5—H5E	109.5	C19—C20—H20B	109.5
H6A—C6—H6B	109.5	C19—C20—H20C	109.5
H6A—C6—H6C	109.5	H20A—C20—H20B	109.5
H6B—C6—H6C	109.5	H20A—C20—H20C	109.5
C7—C6—H6A	109.5	H20B—C20—H20C	109.5
C7—C6—H6B	109.5		
			
Ni1—O1—C7—C6	171.5 (2)	C7—C8—C9—O2	3.4 (5)
Ni1—O1—C7—C8	−8.9 (4)	C7—C8—C9—C10	−174.4 (3)
Ni1—O2—C9—C8	13.7 (4)	C11—N2—C17—N1	0.0 (3)
Ni1—O2—C9—C10	−168.4 (2)	C11—N2—C17—N3	179.3 (3)
Ni1—O3—C1—C2	6.1 (4)	C11—C12—C13—C14	−1.6 (5)
Ni1—O3—C1—C5	−173.9 (2)	C12—N1—C17—N2	0.2 (3)
Ni1—O4—C3—C2	−5.3 (4)	C12—N1—C17—N3	−179.1 (3)
Ni1—O4—C3—C4	175.92 (19)	C12—C11—C16—C15	−0.3 (4)
Ni1—N2—C11—C12	179.46 (17)	C12—C13—C14—C15	1.4 (5)
Ni1—N2—C11—C16	0.1 (4)	C13—C14—C15—C16	−0.8 (5)
Ni1—N2—C17—N1	−179.69 (17)	C14—C15—C16—C11	0.1 (5)
Ni1—N2—C17—N3	−0.4 (4)	C16—C11—C12—N1	179.8 (2)
O1—C7—C8—C9	−6.1 (5)	C16—C11—C12—C13	1.1 (4)
O3—C1—C2—C3	2.2 (5)	C17—N1—C12—C11	−0.3 (3)
N1—C12—C13—C14	−180.0 (3)	C17—N1—C12—C13	178.2 (3)
N2—C11—C12—N1	0.4 (3)	C17—N2—C11—C12	−0.2 (3)
N2—C11—C12—C13	−178.3 (3)	C17—N2—C11—C16	−179.6 (3)
N2—C11—C16—C15	179.0 (3)	C18—N1—C12—C11	−176.5 (3)
C1—C2—C3—O4	−2.6 (5)	C18—N1—C12—C13	2.0 (5)
C1—C2—C3—C4	176.1 (3)	C18—N1—C17—N2	176.4 (3)
C5—C1—C2—C3	−177.7 (3)	C18—N1—C17—N3	−3.0 (4)
C6—C7—C8—C9	173.5 (3)		

### (2-Amino-1-methylbenzimidazole-κN3)aquabis(4-oxopent-2-en-2-olato-κ2O,O')nickel(II) ethanol monosolvate Hydrogen-bond geometry (Å, º)

**Table:**

*D*—H···*A*	*D*—H	H···*A*	*D*···*A*	*D*—H···*A*
N3—H3*B*···O6^i^	0.86	2.09	2.897 (3)	156
O5—H5*A*···O4^ii^	0.85	1.99	2.773 (3)	152
O5—H5*B*···O2^ii^	0.96	1.86	2.777 (3)	160
O6—H6···O1	0.82	2.01	2.811 (4)	166

Symmetry codes: (i) −*x*, −*y*+1, −*z*+1; (ii) −*x*+1, −*y*, −*z*+1.

## References

[bb1] Ablo, E., Dosso, O., Coulibaly, B., Adingra, K. F., Coulibaly, P. M. A., Achi, A. P. & Coulibali, S. (2023). *Adv. Biol. Chem*. **13**, 182–191.

[bb3] Anzenhofer, K. & Hewitt, T. G. (1971). *Z. Kristallogr. Cryst. Mater.* **134**, 54–68.

[bb4] Bansal, Y. & Silakari, O. (2012). *Bioorg. Med. Chem.* **20**, 6208–6236. 10.1016/j.bmc.2012.09.013 23031649

[bb5] Bhrigu, B., Siddiqui, N., Pathak, D., Alam, M. S., Ali, R. & Azad, B. (2012). *Acta Pol. Pharm.* **69**, 53–62. 22574507

[bb6] Binnemans, K. (2005). *Rare-Earth Beta-Diketonates*. In *Handbook on the Physics and Chemistry of Rare Earths*, edited by P. Vitalij, & J.-C. Bunzli, pp. 107–272. Amsterdam: Elsevier.

[bb7] Bourhis, L. J., Dolomanov, O. V., Gildea, R. J., Howard, J. A. K. & Puschmann, H. (2015). *Acta Cryst.* A**71**, 59–75. 10.1107/S2053273314022207 PMC4283469 25537389

[bb8] Caminati, V. & Grabow, J. U. (2006). *J. Am. Chem. Soc.* **128**, 854–857. 10.1021/ja055333g 16417375

[bb26] Cramer, R. E., Cramer, S. W., Cramer, K. F., Chudyk, M. A. & Seff, K. (1977). *Inorg. Chem.* **16**, 219–223. 10.1021/ic50178a601 22668244

[bb9] Desai, K. G. & Desai, K. R. (2006). *Bioorg. Med. Chem.* **14**, 8271–8279. 10.1016/j.bmc.2006.09.017 17035035

[bb10] Dolomanov, O. V., Bourhis, L. J., Gildea, R. J., Howard, J. A. K. & Puschmann, H. (2009). *J. Appl. Cryst.* **42**, 339–341.

[bb11] Duan, Y. Y., Wu, D. F., Chen, H. H., Wang, Y. J., Li, L., Gao, H. L. & Cui, J. Z. (2022). *Polyhedron*, **225**, 116070.

[bb12] Elnima, E. I., Zubair, M. Yu. & Al-Badr, A. A. (1981). *Antimicrob. Agents Chemother.* **19**, 29–32. 10.1128/aac.19.1.29 PMC181352 7247359

[bb13] Groom, C. R., Bruno, I. J., Lightfoot, M. P. & Ward, S. C. (2016). *Acta Cryst.* B**72**, 171–179. 10.1107/S2052520616003954 PMC4822653 27048719

[bb27] Hämmerling, S., Mann, L., Steinhauer, S., Kuntze-Fechner, M. W., Radius, U. & Riedel, S. (2018). *Z. Anorg. Allg. Chem.* **644**, 1047–1050.

[bb14] Jain, A., Sharma, R. & Chaturvedi, S. S. (2013). *Med. Chem. Res.* **22**, 4622–4632.

[bb15] Jongh, L. A. de, Strasser, C. E., Raubenheimer, H. G. & Cronje, S. (2009). *Polyhedron*, **28**, 3635–3641.

[bb16] Kabi, A. K., Sravani, S., Gujjarappa, R., Garg, A., Vodnala, N., Tyagi, U. & Malakar, C. C. (2022). *Nanostructured Biomaterials: Basic Structures and Applications*, edited by B. P. Swain, pp. 351–378. Dordrecht: Springer.

[bb17] Keri, R. S., Hiremathad, A., Budagumpi, S. & Nagaraja, B. M. (2015). *Chem. Biol. Drug Des.* **86**, 19–65. 10.1111/cbdd.12462 25352112

[bb18] Kido, J., Nagai, K. & Ohashi, Y. (1990). *Chem. Lett.* **19**, 657–660.

[bb19] Kuzmina, N. P. & Eliseeva, S. V. (2006). *Russ. J. Inorg. Chem*. **51**, 73–88.

[bb20] Li, G. R., Liu, J., Pan, Q., Song, Z. B., Luo, F. D., Wang, S. R., Zhang, X. L. & Zhou, X. (2009). *Chem. Biodivers.* **6**, 2200–2208. 10.1002/cbdv.200800281 20020452

[bb21] Marinescu, M. (2023). *Antibiotics*, **12**, 1220. 10.3390/antibiotics12071220 PMC10376251 37508316

[bb22] Mathew, B., Suresh, J. & Anbazhagan, S. (2016). *J. Saudi Chem. Soc*. **20**, S132–S139.

[bb2] Morcoss, M. M., Abdelhafez, E. S. M. N., Ibrahem, R. A., Abdel-Rahman, H. M., Abdel-Aziz, M. & Abou El-Ella, D. A. (2020). *Bioorg. Chem.* **101**, 103956. 10.1016/j.bioorg.2020.103956 32512267

[bb23] Pathare, B. & Bansode, T. (2021). *Results Chem*. **3**, 100200.

[bb24] Ramanatham, V., Sanjay, D. V., Bobba, V. S. K., Umesh, N. B., Shekhar, B. B. & Uday, K. M. (2008). *Eur. J. Med. Chem.* **43**, 986–995.

[bb25] Rigaku OD (2020). *CrysAlis PRO*. Agilent Technologies Ltd, Yarnton, England.

[bb28] Shabana, K., Salahuddin, Mazumder, A., Singh, H., Kumar, R., Tyagi, S. & Kumar Yadav, R. (2023). *ChemistrySelect*, **8**, e202300209.

[bb29] Sheldrick, G. M. (2015). *Acta Cryst.* C**71**, 3–8.

[bb30] Smith, K. T., Young, S. C., DeBlasio, J. & Hamann, C. S. (2016). *J. Chem. Educ.* **93**, 790–794.

[bb31] Spackman, P. R., Turner, M. J., McKinnon, J. J., Wolff, S. K., Grimwood, D. J., Jayatilaka, D. & Spackman, M. A. (2021). *J. Appl. Cryst.* **54**, 1006–1011. 10.1107/S1600576721002910 PMC8202033 34188619

[bb32] Tajane, P. S. & Sawant, R. L. (2022). *Int. J. Health Sci.* **6**, 7169–7179.

[bb33] Thomas, M. H. (2001). *2,4-Pentandione*. In *Encyclopedia of Reagents for Organic Synthesis*. Chichester: Wiley.

[bb34] Tighadouini, S., Roby, O., Mortada, S., Lakbaibi, Z., Radi, S., Al-Ali, A. & Warad, I. (2022). *J. Mol. Struct.* **1247**, 131308.

[bb35] Townsend, L. B., Devivar, R. V., Turk, S. R., Nassiri, M. R. & Druck, J. K. (1995). *J. Med. Chem.* **38**, 4098–4105. 10.1021/jm00020a025 7562945

[bb36] Venkatesan, P., Thamotharan, S., Ilangovan, A., Liang, H. & Sundius, T. (2016). *Spectrochim. Acta A Mol. Biomol. Spectrosc.* **153**, 625–636. 10.1016/j.saa.2015.09.002 26452098

[bb37] Zheleznova, L., Sliusarchuk, L., Rogovtsov, O., Kuleshov, S. & Trunova, O. (2021). *Mol. Cryst. Liq. Cryst.* **717**, 14–23.

